# From Interaction to Co-Association —A Fisher *r*-To-*z* Transformation-Based Simple Statistic for Real World Genome-Wide Association Study

**DOI:** 10.1371/journal.pone.0070774

**Published:** 2013-07-29

**Authors:** Zhongshang Yuan, Hong Liu, Xiaoshuai Zhang, Fangyu Li, Jinghua Zhao, Furen Zhang, Fuzhong Xue

**Affiliations:** 1 Department of Epidemiology and Health Statistics, School of Public Health, Shandong University, Jinan, China; 2 Shandong Provincial Institute of Dermatology and Venereology, Shandong Academy of Medical Science, Jinan, China; 3 MRC Epidemiology Unit and Institute of Metabolic Science, Cambridge, United Kingdom; University of California, Irvine, United States of America

## Abstract

Currently, the genetic variants identified by genome wide association study (GWAS) generally only account for a small proportion of the total heritability for complex disease. One crucial reason is the underutilization of gene-gene joint effects commonly encountered in GWAS, which includes their main effects and co-association. However, gene-gene co-association is often customarily put into the framework of gene-gene interaction vaguely. From the causal graph perspective, we elucidate in detail the concept and rationality of gene-gene co-association as well as its relationship with traditional gene-gene interaction, and propose two Fisher *r-to-z* transformation-based simple statistics to detect it. Three series of simulations further highlight that gene-gene co-association refers to the extent to which the joint effects of two genes differs from the main effects, not only due to the traditional interaction under the nearly independent condition but the correlation between two genes. The proposed statistics are more powerful than logistic regression under various situations, cannot be affected by linkage disequilibrium and can have acceptable false positive rate as long as strictly following the reasonable GWAS data analysis roadmap. Furthermore, an application to gene pathway analysis associated with leprosy confirms in practice that our proposed gene-gene co-association concepts as well as the correspondingly proposed statistics are strongly in line with reality.

## Introduction

Since the first successful genome-wide association study (GWAS) for age-related macular degeneration published in 2005 [1], numerous loci associated with complex human disease or traits have been identified. Despite high expectations, the genetic variants identified by GWAS, though providing valuable insights into genetic architecture, generally only account for a small proportion of the total heritability for complex disease [2], [3]. Potential explanations may include underestimation of the effects of alleles identified, the existence of gene-gene joint effects, the contribution of rare variation, the possibility that inherited epigenetic factors lead to resemblance between relatives, and possible overestimation of heritability of the complex traits [2], [3], [4], [5]. Moreover, recent technological advances in high-throughput sequencing platforms enables the acquisition of genomic data at unprecedented speed and amounts, in fact, the capacity to generate the data greatly outpaces our ability to analyze and interpret. It is, therefore, quite desirable to further develop more efficient data mining strategy to extract more information from huge GWAS data, rather than put them aside.

**Figure 1:**
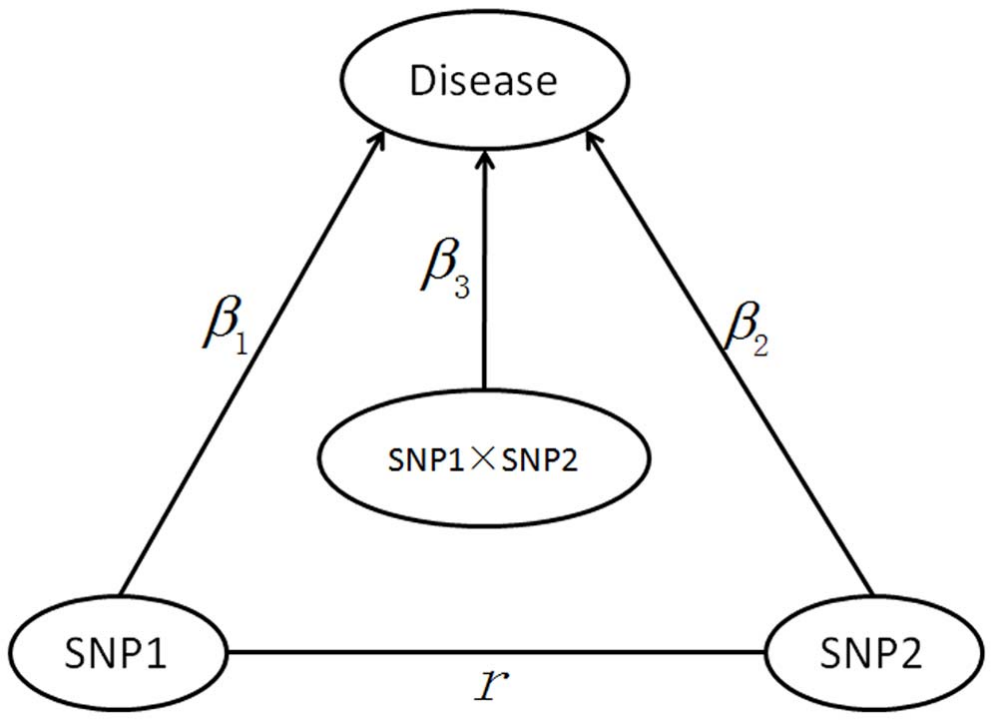
A causal graph framework for two SNPs affected the disease. 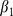
 and 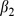 represents main effects, 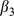 denotes the traditional interaction, the nondirectional arc between SNP1 and SNP2 (correlation *r*) indicated that the two variables are associated for reasons other than affecting one another.

Among the data analysis demand, one major issue refers to the joint effects of multiple genes contributing to the interested disease or trait. The joint effect of two genes included their main effects and co-association. We have proposed the concept of gene-gene co-association in previous studies [6], [7], which refers to the extent to which the joint effect of two genes on disease (or trait) differs from the main effects of each gene. Traditional methods customarily put gene-gene co-association into the framework of gene-gene interaction. To determine the presence of interactions between two genes, regression-based approaches are still regarded as the most natural first-line approach, though some alternative methods have been developed [8], [9], [10], [11], [12], [13], [14], [15]. A product term is usually added to the logistic regression model (LRT) 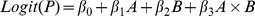 for the popular case-control design in GWAS, which implies a nearly independence assumption, at least not much correlation, between gene A and gene B for inferring the interaction (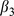). Nevertheless, one common sense is that the development of most common diseases is attributed to complex gene network system. Genes (or SNPs) are often correlated with each other in the following situations: 1) genes (or SNPs) within pathways or networks contributing to a disease; 2) SNPs with linkage disequilibrium (LD) located in two or more linked genes within one chromosome; 3) SNPs with LD in one gene. Hence the above assumption is rarely satisfied. It will be inevitable to lose efficiency using LRT blindly when high correlation existed between SNPs. Actually, the genetic pathway or network, even SNPs co-association within one high LD genome region, can be deemed as a graph and studies should be conducted under graphical framework [16]. Specifically, taking 2 SNPs for simplicity, from a causal diagram perspective (Fig. 1 in Methods), suppose the main effects for SNP1 and SNP2 are 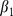 and 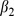 respectively, and the correlation between them is *r*, which is usually far away from zero (e.g., SNPs with LD or two SNPs within one pathway). Then the total effects for SNP1 and SNP2 will be 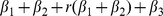, and the term (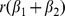) is obviously attributed to the correlation between the two SNPs, which would not be detected efficiently by LRT.

The argument is that, for two genes, the extent to which the joint effects differs from the main effects (i.e. gene-gene co-association), are not only refer to the traditional interaction (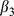) but the correlation between two genes (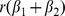). While traditional LRT only provide one way to identify the part under the nearly independent condition, with less power for the left attributed to the correlation. To solve this problem, in the context of a standard case-control design, three gene-based statistics, CCU [6], KCCU [7] and PLSPM-based statistic [17], have been developed in our former work based on the difference of correlation of two genes between cases and controls. Actually, similar idea has already been employed to develop new statistics recently [9], [11], [12]. However, these statistics do not yet jump out from the scope of gene-gene interaction. Particularly, the statistics recently proposed by Rajapakse [11] will be invalid when heavy multicollinearity (strong LD) between SNPs existed as the algorithm needs the computation of the inverse of covariance matrix. For GWAS in practice, at least five aspects for gene-gene co-association should be considered: 1) the theory basis and rationality; 2) efficient and robust statistics to detect it; 3) simple and universally accessible statistics, just like Armitage trend test [18]; 4) the acceptable false positive rate in real world GWAS; 5) the feasibility for computation challenge.

Although various novel statistics for gene-gene interaction or gene-gene co-association have been proposed few of them are successfully used in real world full GWAS data analysis. This is not only due to their elusive statistical model for general geneticist and epidemiologist, but their unrealistic computation burden attributed to some non-parametric methods (e.g., bootstrap or permutation). In this paper, the concept and rationality of gene-gene co-association is elucidated by a simple causal graph. Based on the difference of correlation of two genes between cases and controls, two simple statistics (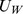 and 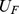) for detecting gene-gene co-association are proposed using Fisher *r*-to-*z* transformation [19], [20]. The former was constructed according to the asymptotic distribution theory of the empirical product-moment correlation coefficient for counting variables [21], while the latter is developed by empirically calibrating Fisher *r*-to-*z* transformation-based simple statistic[19], [20]. Various simulation studies are firstly conducted to assess the type I error rate and power, and to clarify the relationship between gene-gene co-association and gene-gene interaction. And then simulations are carried out to evaluate whether the proposed statistics can be affected by strong LD between SNPs. Furthermore, based on the experimental strategy of gain-of-function in functional genomics [22], [23], simulations are performed by mimicking real world GWAS roadmap to assess their false positives. Finally, we analyze a GWAS real data from a plausible biologic network underlying susceptibility to leprosy [24], and the computation time is also reported.

## Methods

### Fisher *r*-to-*z* transformation-based statistics

For GWAS in case-control design, SNP1 and SNP2 denote the two markers, In the framework of causal graph (Fig. 1), no matter whether they are independent or correlated (within same pathway or with LD between them), the total effects for SNP1 and SNP2 can be illustrated by 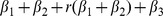 the co-association between them can be defined as 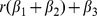, where 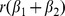 denote the effect on the disease attributed to the correlation, and 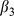 the part under independence condition and often be detected by LRT. Let 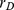 denote the sample correlation coefficient between SNP1 and SNP2 among cases, and 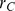 between them among controls. We use 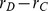 to measure the co-association between the two SNPs contributing to the disease. A Fisher *r-to-z* transformation was proposed earlier for testing the difference between two correlation coefficients [19], [20]. This transformation was done to 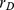 and 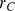, i.e. 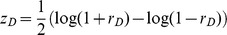 and 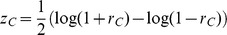, furthermore, Wellek and Ziegler [21] have derived the asymptotic distribution of the empirical correlation for counting variables, our proposed statistic 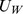 for detecting gene-gene co-association was defined as
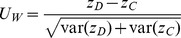
Where 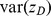 and 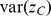 denote the variance estimator from their work. Although 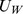 is theoretically accurate, the variance formula in the denominator cannot be obtained quickly since it needs the estimation of population frequencies for various combinations of two specific loci, and we may have to compute it one SNP pairs one time, which is inadvisable for enormous real world GWAS data. Therefore, it is critical to develop a further simpler and more efficient statistic to improve the feasibility and practicability. Fisher has provided a well-known statistic for comparing the correlation coefficients from two samples for data from a bivariate normal distribution, which can be used to detect gene-gene co-association by
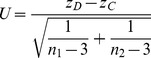
where 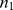 and 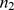 represent the sample size for case and control respectively. Simulations (data not shown) show this simple statistic can have good performance in gene-gene co-association detection for SNPs without much correlation. Nevertheless, when the correlation between SNP pairs is high, the statistic is normal but with variance far from 1 under the null hypothesis. Therefore, an empirically calibrated statistic 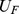 was further proposed as
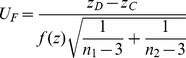
where 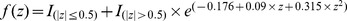, and 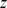 is the corresponding Fisher transformation for pooled sample correlation between SNP1 and SNP2. 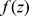 is obtained by empirical simulation based on the fitted functional relationship between correlation coefficients and their variance. Under the additive genetic model, using 88 SNP pairs with various correlation chosen from simulated 100 SNPs on chromosome 21 for 100000 individuals (Fig. S1 for scatter plot), we get the empirical variance formula (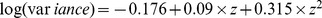, 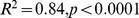), where linear regression is applied by treating the logarithm of empirical variance as response variable, and 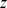 and 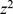 as independent variables. Bearing in mind to correct the variance when the correlation is high, we propose the aforementioned 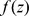.

### Simulation

Our first series of simulation studies were designed to evaluate the type I error rate and power, to clarify the relationship between gene-gene co-association and gene-gene interaction, and to compare the performance of 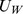, 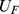 and LRT under different sample size, main effects, co-association effect (including the following three scenarios: co-association under nearly independent condition between gene A and gene B, co-association only caused by correlation between gene A and gene B and co-association caused by both correlation and independent term A
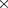
B between gene A and gene B; abbreviate to Type I co-association, Type II co-association and Type III co-association). We chose the first 1000 SNPs on chromosome 21 to simulate genotypes based on HapMap phase II CEU data, and a large population of 100000 individuals was obtained via the software Hapgen2 [25]. Case-control status was generated from a logistic regression 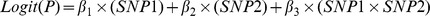, where SNP1 and SNP2, correlated with coefficient *r*, were causal SNPs chosen from the 1000 SNPs so that the MAF can always be kept about 20% and 30% respectively. Three different scenarios were considered (Fig. 1). 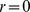, 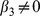 indicated the case that Type I co-association, 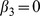, 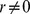 for Type II co-association, and 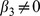, 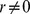 for Type III co-association. Three different main effects were set to make our simulations more practical, no marginal effects (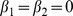), one marginal effect (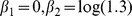), and two marginal effects (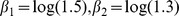). Different 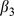 and 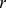 are specified to evaluate the type I error (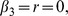 or 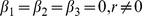) and power. A total of 3000 simulations were repeated for each scenario, and we randomly sample 3000 individuals from the whole 100000 population for each simulation.

For gene-gene co-association, it might give rise to an illusion that SNP pairs would be detected powerfully as long as they were highly correlated (e.g. high LD). Therefore, our second series of simulation studies were devoted to evaluate whether the proposed statistics can be affected by strong LD between SNPs. Two neighbored genes on chromosome *17q21* (*ZPBP2* gene and *GSDMB* gene (Fig. S2 for LD plot), which has been confirmed to be associated with asthma [26], [27], were chosen to simulate genotypes similarly as the aforementioned design. First, the 7^th^ SNP (rs4795400, MAF = 46%) on *GSDMB* gene was defined as the causal SNP with various odds ratio (1.1 to 1.5), and the co-association between the 5^th^ SNP (rs2290400) and 9^th^ SNP (rs7216389) was detected. Second, the 3^th^ SNP (rs12150079, MAF = 26%) on *ZPBP2* gene and the 5^th^ SNP (rs2290400, MAF = 48%) on *GSDMB* gene were specified as causal SNPs, and we fixed odds ratio 1.3 for the second causal SNP, while ranging the odds ratio of the first causal SNP from 1.1 to 1.5. The co-association between the 1^th^ SNP (rs11557466) on *ZPBP2* gene and the 9^th^ SNP (7216389) on *GSDMB* gene was detected. We randomly sample 6000 individuals for each simulation (totally 3000 simulations).

We designed the third series of simulation studies by mimicking real world GWAS roadmap to assess their false positives. Similar as above, the first 1000 SNPs on chromosome 1 were chosen to simulate genotypes. Four situations were considered: two neighbored SNPs within one gene, two SNPs located in two linked exons within one gene, two SNPs located in two linked genes within one chromosome, and two SNPs located in two genes within one pathway from two different chromosomes. For the first situation, we chose the 9^th^ (rs11030107, MAF = 31%) and 15^th^ SNPs (rs10835211, MAF = 30%) from *BDNF* gene (totally 19 SNPs) on chromosome 11 as the causal SNPs with correlation 0.96, then we embeded the gene into the 1000 SNPs on chromosome 1 generated above to mimic 500000 SNP pairs. Three independent sample with sample size 3000, 3000 and 6000, were generated to mimic the GWAS roadmap and to see whether the three samples reported the same false positive SNP pairs under 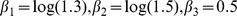. We aim to see how many false positives emerge under the premise that the true causal SNP pairs have been discovered. SNP pairs with *P*-value less than that for the true causal SNP pairs, will be recorded as false positives if they are not located within the *BDNF* gene. The idea behind this design stems from the gain-of-function technique which is usually taken to study the function of a gene [22], [23]. Here the *BDNF* gene can be deemed as the gain-of-function causal variants that affect the function of specified protein, then leading to the final disease. In a similar vein, the 1^th^ (rs7124442, MAF = 36.7%) and 18^th^ SNPs (rs1013402, MAF = 35.9%) with correlation 0.95 for the second situation, the 5^th^ SNP (rs12936231, MAF = 31%) from *ZPBP2* gene and 9^th^ SNP (rs7216389, MAF = 30%) from the *GSDMB* gene from chromosome *17q21* for the third situation, and the 51^th^ (rs12111180, MAF = 31%) SNP from *PARK2* gene on chromosome 6 and 60^th^ SNP from the *LRRK2* gene (rs11564205, MAF = 30%) on chromosome 12 within the pathway (conferring susceptibility to leprosy [24]) for the fourth situation.

### Application

Based on the GWAS of leprosy [24], using Ingenuity Pathways Analysis knowledge database (Ingenuity Systems), a plausible biologic network underlying susceptibility to leprosy was created for depicting the functional relationship between the identified five susceptibility genes (together with five other genes). To further confirm the relationship between the genes in the network, we attempt to detect the co-association between SNP pairs within 9 susceptibility genes (2257 SNPs) by the proposed statistics 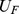 and 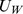, using the initial GWAS data with 706 cases and 514 controls. These 9 genes locate on different chromosomes and totally contained 2257 SNPs (Table S1). Meanwhile, to compare the computation time of all three statistics (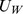, 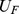 and LRT), a desktop computer (Intel Core i3-2100 with 3.10 GHz CPU using 4 GB of RAM) was used to do calculations by R 2.14.0.

## Results

### Simulation studies

Shown in Table 1 were the estimated type I error rates of LRT and the two proposed statistics under 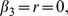 It revealed that all type I error rates were close to nominal level 0.05 as a function of sample sizes. When 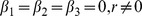, though stable for LRT and 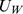, the type I error was slightly higher for 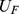 under correlation 0.4 or 0.6 (Table 2). This might be due to 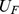 essentially do modifications for high correlation and kept the same as Fisher test when correlation was relatively small.

**Table 1:** Type I error for three statistics without correlation and interaction.

	LRT		*U_W_*		*U_F_*	
Sample size	Type^a^	Type^b^	Type^a^	Type^b^	Type^a^	Type^b^
1000	0.050	0.050	0.047	0.050	0.051	0.051
2000	0.046	0.049	0.049	0.047	0.052	0.047
3000	0.052	0.055	0.055	0.054	0.053	0.053
4000	0.050	0.049	0.054	0.049	0.050	0.049
5000	0.050	0.046	0.055	0.050	0.047	0.053

a For case with one main effects (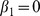, 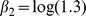),

b For case with two main effects (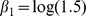, 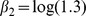).

**Table 2:** Type I error for three statistics without main effects under sample size 3000 and 5000.

	LRT		*U_W_*		*U_F_*	
Correlation coefficient	3000	5000	3000	5000	3000	5000
0.2	0.050	0.050	0.040	0.056	0.055	0.058
0.4	0.046	0.049	0.045	0.055	0.076	0.071
0.6	0.052	0.055	0.048	0.044	0.073	0.066
0.8	0.050	0.049	0.058	0.056	0.058	0.056

**Figure 2:**
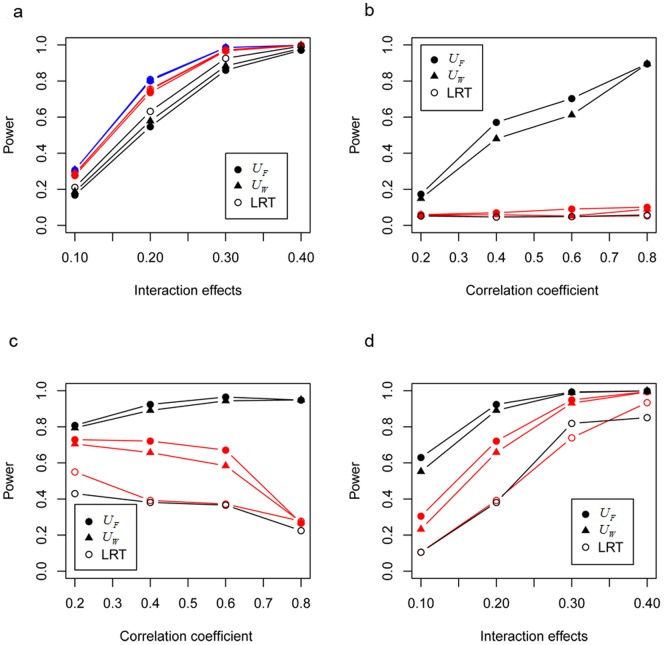
Simulations for clarifying the relationship between co-association and interaction. a for Type I co-association; b for Type II co-association; c for Type III co-association given fixed interaction effect 0.2 and different correlation; d for Type III co-association given fixed correlation 0.4 and different interaction effects. The case with no main effects (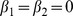), one main effects (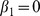, 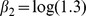) and two main effects (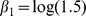, 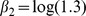) are shown by blue, red and black lines respectively.

Fig. 2a showed the performance with 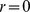 and 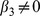 (Type I co-association) when 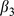 was set to be 0.1, 0.2, 0.3, 0.4. It indicated that the power of all three methods increase monotonically with the interaction effect (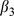). Both 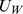 and 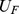 had almost comparable power with LRT, which was the gold standard in this case. Shown in Fig. 2b was the power for the situation of Type II co-association (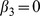 and 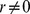), we set *r* to be 0.2, 0.4, 0.6, 0.8. The power for the two proposed statistics kept relatively high and increased slowly as the correlation coefficient (*r*) increases, while LRT had completely lost the power. Furthermore, the power for the situation with two main effects was higher than that for the situation with only one main effect. Fig. 2c showed the power for the situation of Type III co-association with fixed 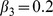, the power for LRT was relatively lower and decreased as the correlation increases, this might be partly due to the high variance for 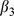 attributed to the increasing correlation. The proposed two statistics had higher power. For the situation with one main effect, though 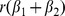 increased, the power for co-association decreased as correlation increases from 0.2 to 0.8, which might be due to the power for detecting the interaction 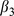 decreased as correlation increased. Fig. 2d presented the power for the situation of Type III co-association under fixed correlation 0.4. The power for all statistics increased as the interaction effect increase from 0.1 to 0.4, and the two proposed statistic always had higher power than LRT. All results illustrated that, under medium correlation, the power for 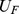 seems a little higher than that for 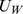.

**Figure 3:**
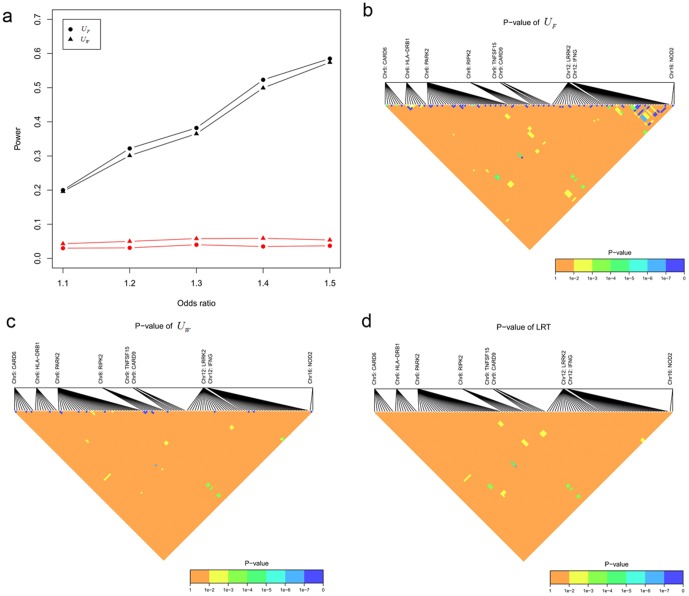
Simulation for assessing the effect of LD and application to gene pathway analysis associated with leprosy, a simulation results for assessing the effect of LD, red line for the case with one causal SNP, black line for case with two causal SNPs; b pathway analysis by 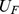
 **; c pathway analysis by** 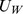 **; d pathway analysis by LRT.**

Fig. 3a showed the results for the second series of simulation studies, the 5^th^ and 9^th^ SNP on *GSDMB* gene, though had some indirect main effects due to LD with the causal SNP (7^th^ SNP), showed no co-association between them, indicating that no power gained when the indirect main effects of two SNPs originated from only one causal variant. While for the situation with two causal SNPs, the 1^th^ SNP on *ZPBP2* gene and the 9^th^ SNP on *GSDMB* gene, with each having indirect main effect due to LD with the causal SNPs (the 3^th^ SNP on *ZPBP2* gene and the 5^th^ SNP on *GSDMB* gene), showed some co-association between them. These elucidate that the proposed statistics cannot be affected by LD, and the co-association indeed represented nothing but the effect contributing to the disease.

Following up the real world GWAS data analysis roadmap (with sample size 3000, 3000, 6000 respectively), the 1000 SNPs on chromosome 1 with nearly 500000 SNP pairs are simulated 100 times. Among the nearly 100*500000 tests under the four situations designed in the third series of simulation, the total false positive rate was about 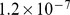 for 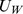, 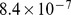 for 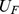 and 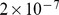 for LRT respectively.

### Real data

Fig. 3b–3d and Table 3 showed the results of gene-gene co-association analysis for 2257 SNPs within 9 susceptibility genes belonging to the pathway associated with leprosy [24]. For ease of visualization, only SNPs within SNP pairs whose *p*-value less than 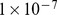 in at least one of the three methods were presented. For SNP pairs in two different genes, all three statistics had similar results. The co-association between *PARK2* and *LRRK2* was detected at 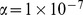 by 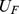 and 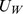, 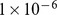 by LRT, the correlation coefficient between the two SNPs of *PARK2* (rs6904305) and *LRRK2* (rs12814017) is 0.13. The co-association between *NOD2* and *IFNG*, *IFNG* and *PARK2* was also detected at 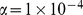 by 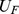 and 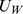, 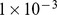 by LRT; between *IFNG* and *CARD6* at 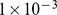 by 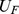, 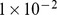 by 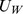
and LRT. The marked genes with self-regulation in the network [24] were also detected by both 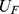 and 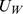 at 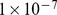 level, while nothing appeared by LRT due to the stronger LD between SNPs within one gene. This indicated that the results from 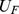 and 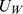 strongly agreed with that from the Ingenuity Pathways Analysis knowledge database, while not from LRT in the framework of traditional gene-gene interaction. In addition, all three statistics showed there was some co-association between IFNG and PARK2 (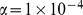), both 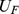 and 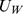 suggest that self-regulation within *LRRK2*, *PARK2*, *TNFSF15*, and *CARD6* may also exist (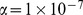), though these were not marked in the network. The computation time for 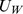 takes nearly 25 hours, and about 28 hours for LRT, while only 3 minutes for statistic 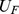 using the same desktop computer (Intel Core i3-2100 with 3.10 GHz CPU using 4 GB of RAM).

**Table 3:** Gene-gene co-association for SNP pairs (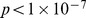) within 9 susceptibility genes belonging to the pathway associated with leprosy.

SNP pairs	Gene	SNP pairs	Gene
rs16869977-rs10512739	CARD6	rs39503-rs447618	RIPK2
rs11744119-rs2271709	CARD6	rs39503-rs411279	RIPK2
rs4245977-rs10473238	CARD6	rs447618-rs411279	RIPK2
rs1815510-rs3177253	HLA-DRB1	rs6470668-rs7815279	RIPK2
rs1822520-rs1404269	PARK2	rs6470668-rs7459577	RIPK2
rs1822520-rs9365252	PARK2	rs7855735-rs1125441	TNFSF15
rs10945765-rs2281403	PARK2	rs10880160-rs1390995	LRRK2
rs11962721-rs10945770	PARK2	rs776421-rs776207	IFNG
rs1789995-rs1789993	PARK2	rs775450-rs775448	IFNG
		rs7186144-rs8043960	NOD2

## Discussion

### The rationality and significance of gene-gene co-association

From the causal graph perspective [16], we elucidated the concept and rationality of gene-gene co-association, and clarify its relationship with the traditional gene-gene interaction. Simulation studies further confirm our viewpoint. Fig. 2a shows that the co-association is almost the same as the interaction in the situation co-association with standalone interaction. Fig. 2b demonstrates that the co-association still exists in the situation co-association without interaction. Fig. 2c and 2d illustrate the situation of Type III co-association, indicating that it will be lost some power when replacing co-association with interaction. Actually, these relationships have also been supported by a gene-gene interaction study [11], though it does not yet jump out from the scope of traditional gene-gene interaction. The statistic they proposed based on the difference of the covariance matrix between cases and controls, showing much power than the LRT, indeed measure the co-association between two genes essentially, rather than their interaction. Specifically, simulation indicated that their proposed statistic showed no power when the two genes only have marginal effect on the disease (case 1 in their work). This is actually the situation of Type II co-association (Fig. 2b in our simulation), the reason why no power emerge is that the two selected gene region (*EXT2* and *LRRC4CX2*) in their simulation are far away from each other and can be considered to be independent. In summary, gene-gene co-association refers to the extent to which the joint effects of two genes differs from the main effects, not only due to the traditional interaction under the nearly independent condition but the correlation between two genes, while the part attributed to the correlation has usually been neglected in traditional interaction model using regression method. Genetically, most diseases are caused by multiple genes acting together through pathways or network that can lead to a common final disease or trait. In practice, when constructing a priori topological structure for establishing genetic networks that contribute to diseases of interest, we often need to test whether significant relationships between any two nodes in such networks exist. It seems more reasonable to solve this by detection for gene-gene co-association rather than traditional interaction.

### Fisher *r*-to-*z* transformation-based simple statistics for gene-gene co-association

Wellek and Ziegler [21] derived the asymptotic distribution of the empirical product-moment correlation coefficient for counting variables. One statistic we here proposed, based on the strict theory from their work, is 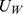. Alternatively, from the feasibility and practicability perspective, we empirically calibrate the traditional Fisher *r*-to-*z* transformation-based simple statistic 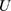 and proposed 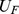, which is not only prone to easy understanding and universally accessible to everyone, but compute fast in practice. Simulation showed that the proposed two statistics are stable, though the type I error of 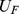 slightly deviates from the nominal level due to the empirical approximation (Table 2). There seems a tradeoff between the accuracy and the computation burden, the theoretical statistic 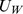 was accurate but with high computation burden, while 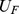 could reduce the computation burden substantially but might lose some accuracy. Both 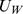 and 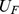 have comparable power with LRT under Type I co-association, while more powerful than LRT under Type II co-association and Type III co-association no matter what the correlation between the SNPs is (Fig. 2). This indicates that the two proposed statistics have good performance for detecting gene-gene co-association. Intuitively, it might give rise to an illusion that the co-association between SNP pairs would be detected powerfully as long as they were highly correlated (e.g. high LD). However, our results illustrate that both 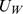 and 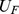 cannot be affected by LD, and co-association indeed represents nothing but the effect contributing to the disease (Fig. 3a). It is important to guard against possible heterogeneity caused by some other covariates (e.g. age, gender, smoking). One possible solution for this is Mantel-Haenszel method, which may suffer small sample size problem when the number of covariates is quite large. Another possible way is to calculate the partial correlation conditional on the covariates in cases and controls respectively.

### The advantages of statistics 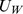 and 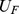 in real world GWAS data analysis

For real world GWAS data analysis, one way to search for co-association (or interaction) is arguably by exhaustive search, which consider all possible pairs of loci and perform the desired co-association test for each pair (e.g. about 500000 SNP pairs for 1000 loci). Therefore, whether one statistic can be used in real world GWAS data analysis depends on two key aspects at least, the acceptable false positive rate and computation burden. Simulation following up the GWAS data analysis roadmap indicates that the false positive rate of the proposed two statistics (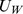 and 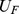) together with LRT are all at about 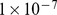 order of magnitude. Also, it indicates that the false positives can be acceptable and control well as long as researchers strictly followed the reasonable GWAS data analysis roadmap. As an example, all three statistics were used to analyze 2257 SNPs (2545896 SNP pairs) within 9 susceptibility genes belonging to the pathway associated with leprosy using a desktop computer (Intel Core i3-2100 with 3.10 GHz CPU using 4 GB of RAM), the computation time for 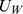 takes nearly 25 hours, and about 28 hours for LRT, while only 3 minutes for statistic 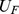, which may be currently the most realistic and feasible statistic.

### Application to gene pathway analysis associated with leprosy

The GWAS for leprosy showed that variants of genes in the NOD2-mediated signaling pathway (which regulates the innate immune response) are associated with susceptibility to infection with *M. leprae*, and a further plausible biologic network was created for highlighting the functional relationship between the susceptibility genes by Ingenuity Systems. In this paper, the co-association analysis between the genes (or SNPs) in this network indicate that the results from 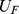 and 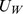 strongly agree with that from the Ingenuity Pathways Analysis knowledge database, while not from LRT in the framework of traditional gene-gene interaction (Fig.3b–3d and Table 3). This further confirm in practice that our proposed gene-gene co-association concept as well as the correspondingly proposed statistics are strongly in line with reality. In addition, the pathway between IFNG and PARK2may also exist as the co-association between them was detected significantly by all three statistics at 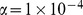 level. So as the self-regulation within LRRK2, PARK2, TNFSF15, and CARD6, since the co-association between multiple SNP pairs within them were also detected significantly by 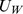 and 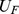 at 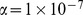. Further replication need to be done to confirm these findings.

### Limitations

One has to realize that the implications of gene-gene interaction are scale-dependent, we here just illustrate gene-gene co-association by comparison with multiplicative interactions in LRT, where the final term expresses a departure from a simple additive model on the logit scale. As one reviewer suggested, we have also assessed the performance for rare variation (MAF<0.05) and found that the type I error is unstable, which suggested that the proposed methods was invalid for rare variation. In addition, although the proposed empirical statistic 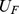 has nearly same performance with the theoretical statistic 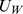, its type I error deviates slightly from the given nominal level (Table 2) after all, this may elevate a little false positive rate. We want to emphasize that different empirical fitness methods may generate different function *f* in the denominator of 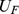, the basic rule for 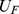, we think, is to improve the computation efficiency and feasibility, meanwhile keep the performance nearly the same as 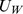.

## Supporting Information

Figure S1 **The scatter plot for the correlation with corresponding empirical variance.**
(TIF)

Figure S2 **The LD plot for two neighbored genes on chromosome 17q21, with the first 8 SNPs belonging to** ***ZPBP2*** **gene and the left belonging to** ***GSDMB*** **gene.**
(TIF)

Table S1 **The location and SNP number for 9 susceptibility genes belonging to the pathway associated with leprosy.**
(DOC)
