# Supplementary figures and images for: From Interaction to Co-Association —A Fisher r-To-z Transformation-Based Simple Statistic for Real World Genome-Wide Association Study
Source: PLoS One. 2013 Jul 29;8(7):e70774. doi: 10.1371/journal.pone.0070774 (PMC3726765; doi:10.1371/journal.pone.0070774)

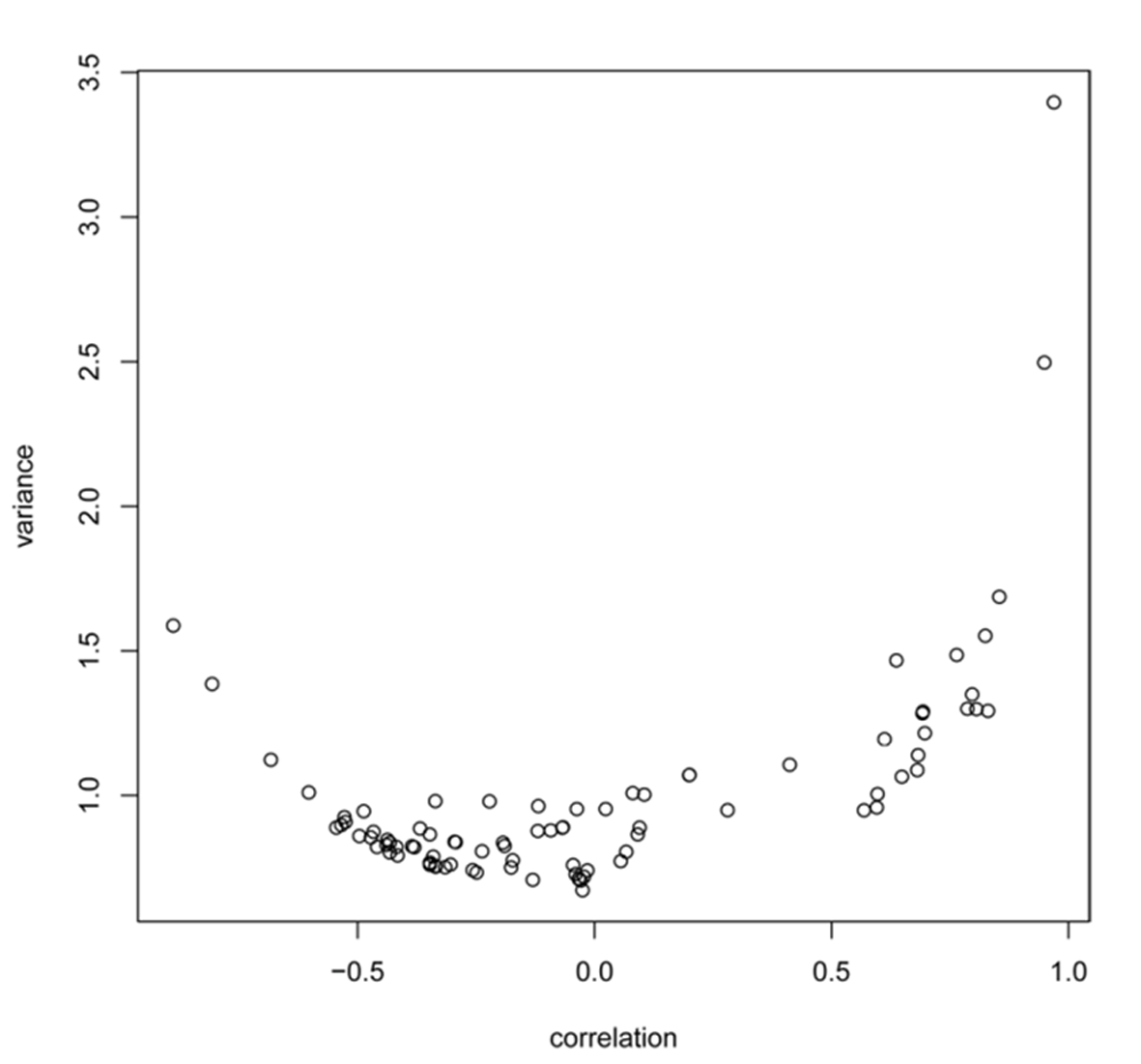

Supplement: Figure S1 — The scatter plot for the correlation with corresponding empirical variance. (TIF) [file pone.0070774.s001.tif]

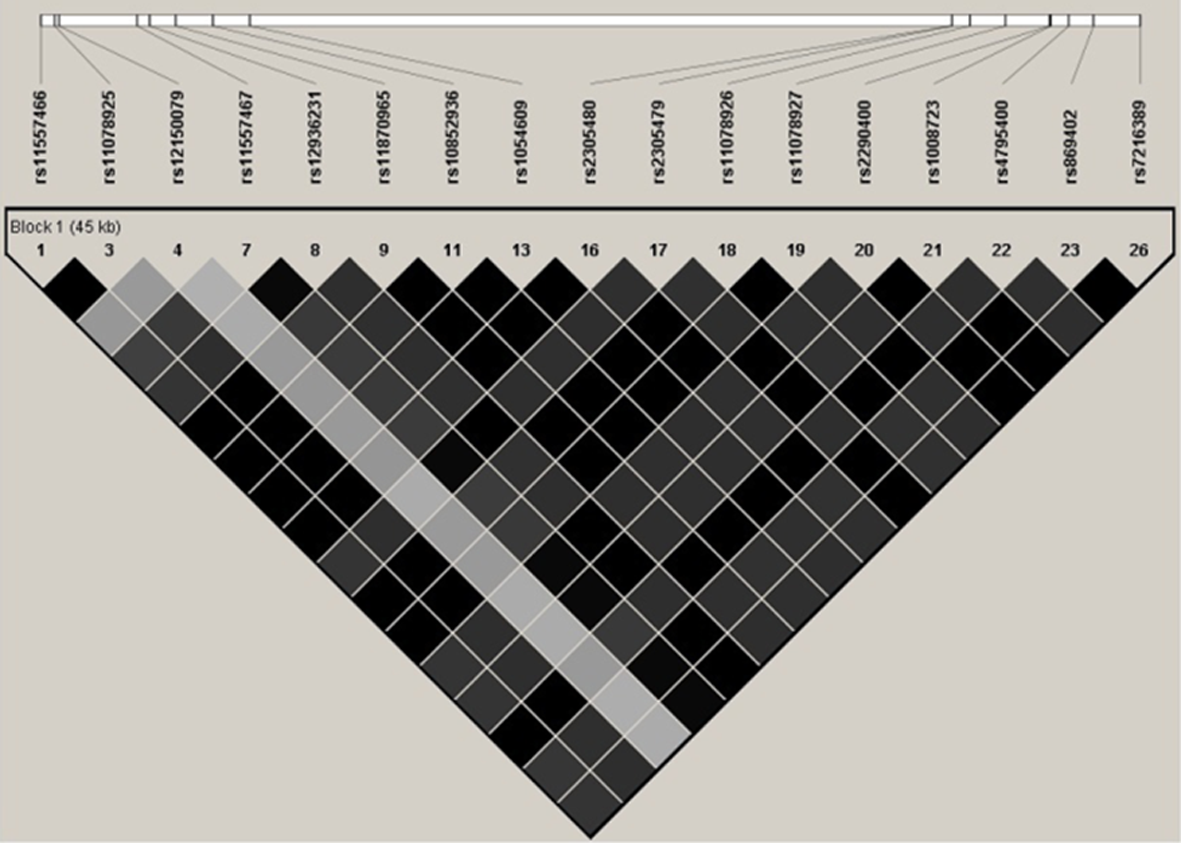

Supplement: Figure S2 — The LD plot for two neighbored genes on chromosome 17q21, with the first 8 SNPs belonging to ZPBP2 gene and the left belonging to GSDMB gene. (TIF) [file pone.0070774.s002.tif]
