# Supplementary material for: From Interaction to Co-Association —A Fisher r-To-z Transformation-Based Simple Statistic for Real World Genome-Wide Association Study
Source: PLoS One. 2013 Jul 29;8(7):e70774. doi: 10.1371/journal.pone.0070774 (PMC3726765; doi:10.1371/journal.pone.0070774)
Supplement: Table S1 — The location and SNP number for 9 susceptibility genes belonging to the pathway associated with leprosy. (DOC) [file pone.0070774.s003.doc]

**Table S1** The location and SNP number for 9 susceptibility genes belonging to the pathway associated with leprosy

| **Gene** | **Gene ID** | **SNP number** | **Chromosome** |
| --- | --- | --- | --- |
| CARD6 | 84674 | 197 | 5 |
| HLA-DRB1 | 3123 | 246 | 6 |
| PARK2 | 5071 | 723 | 6 |
| RIPK2 | 8767 | 181 | 8 |
| TNFSF15 | 9966 | 190 | 9 |
| CARD9 | 64170 | 69 | 9 |
| LRRK2 | 120892 | 225 | 12 |
| IFNG | 3458 | 221 | 12 |
| NOD2 | 64127 | 205 | 16 |
